# Supplementary material for: Epigenetic insights into neuropsychiatric and cognitive symptoms in Parkinson’s disease: A DNA co-methylation network analysis
Source: NPJ Parkinsons Dis. 2025 Mar 2;11:39. doi: 10.1038/s41531-025-00877-5 (PMC11873129; doi:10.1038/s41531-025-00877-5)
Supplement: Supplementary file 1 — Supplementary Figures [file 41531_2025_877_MOESM1_ESM.docx]

**
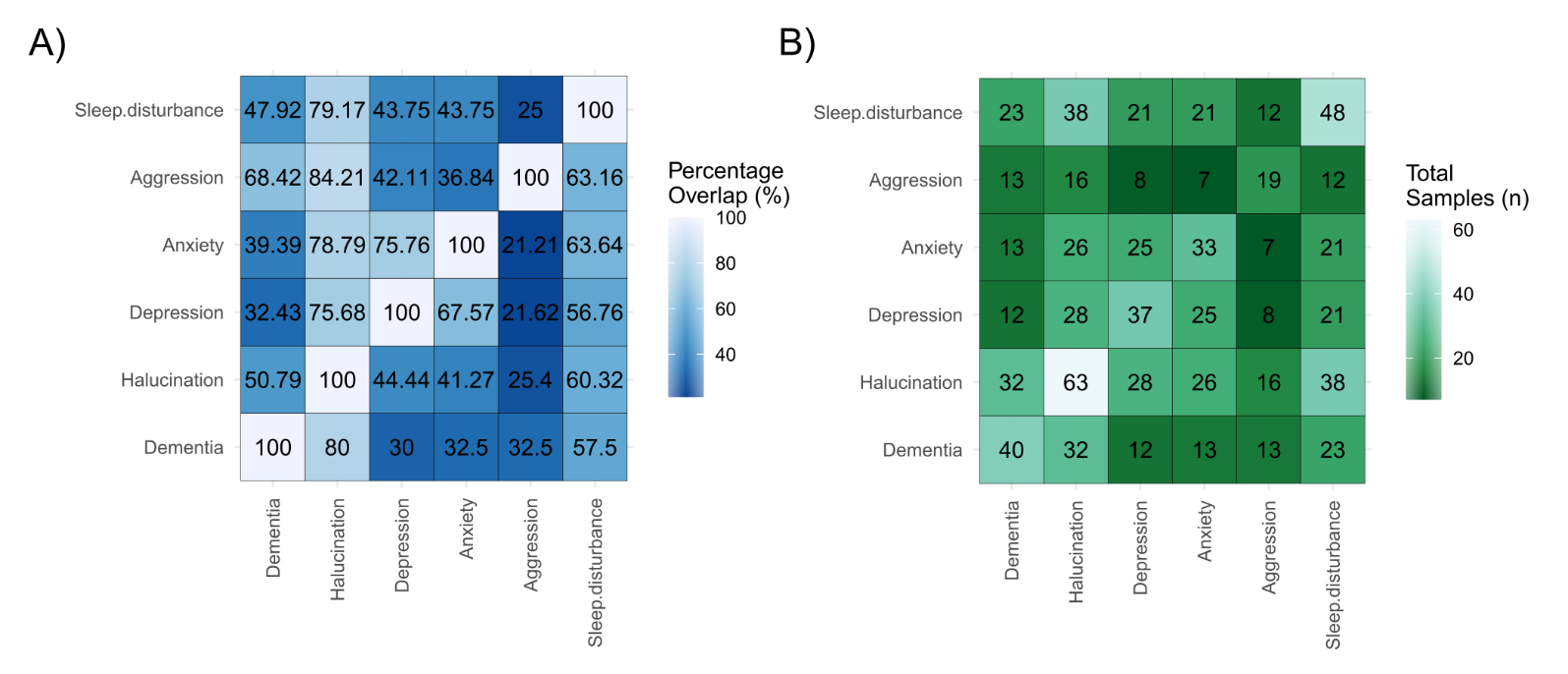
Supplementary Figure 1:** **Symptom Overlap Matrices**. Grids display in correlation matrix format the proportion of overlap in paired symptom presentation. **A)** shows this as a percentage overlap, interpreted as the percentage of samples with symptoms displayed along the Y-axis that also have the symptom along the X-axis. For example, the top left value of 47.92% refers to the percentage of samples with sleep disturbance that also had dementia. **B)** shows the same information but depicted as raw total sample numbers with particular symptom overlap.

**
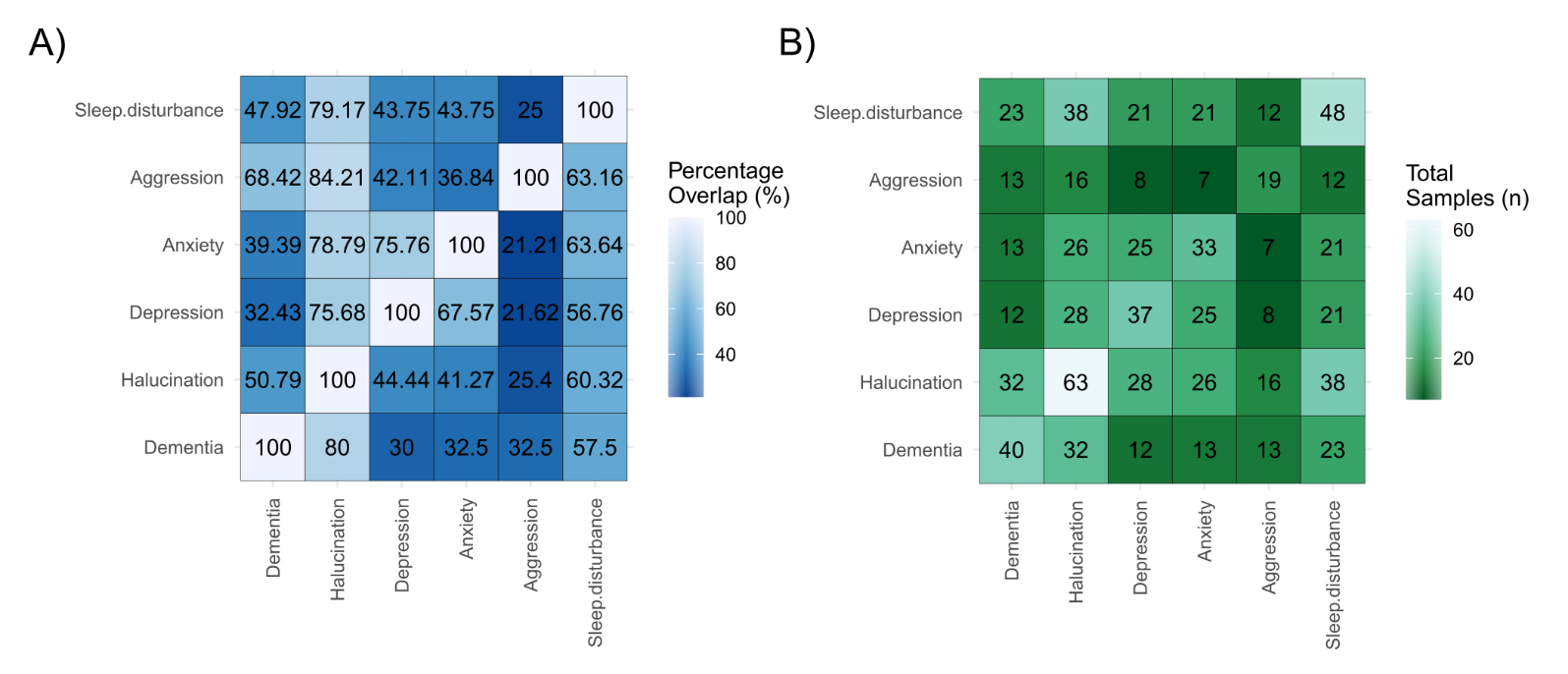
**

**
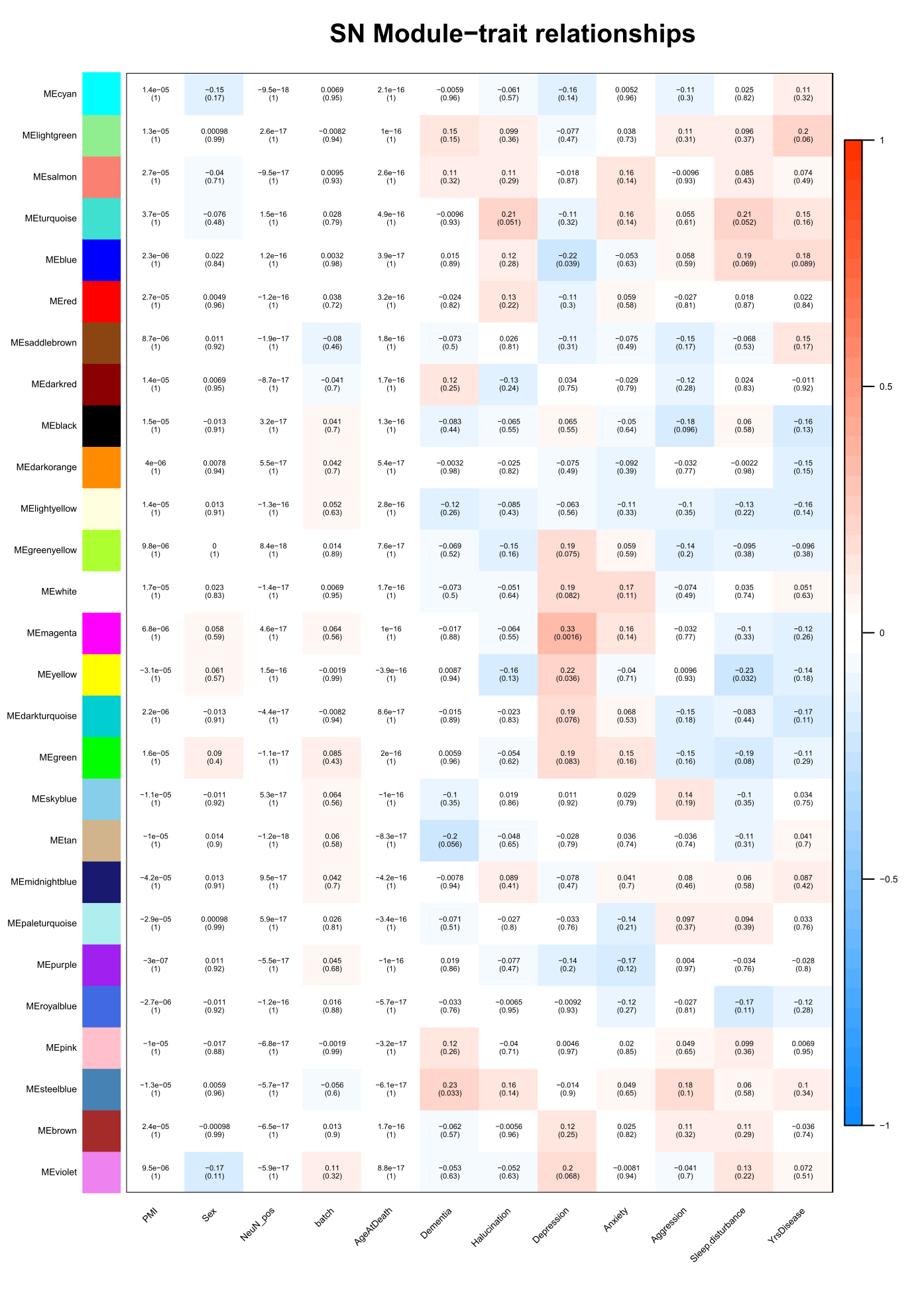
Supplementary Figure 2: Trait-module correlation matrices for the substantia nigra.** Correlations are shown between module eigengenes and traits of interest, with module names (arbitrarily assigned colors) shown along the Y-axis. Correlation estimates are reported, with p-values in parentheses. Grids are colored by correlation estimates. Abbreviations: PMI: Post mortem interval, NeuN_pos: Predicted NeuN+ proportion, batch: Processing batch value, AgeAtDeath: Years of age at death, YrsDisease: Years between diagnosis of PD and death.

**
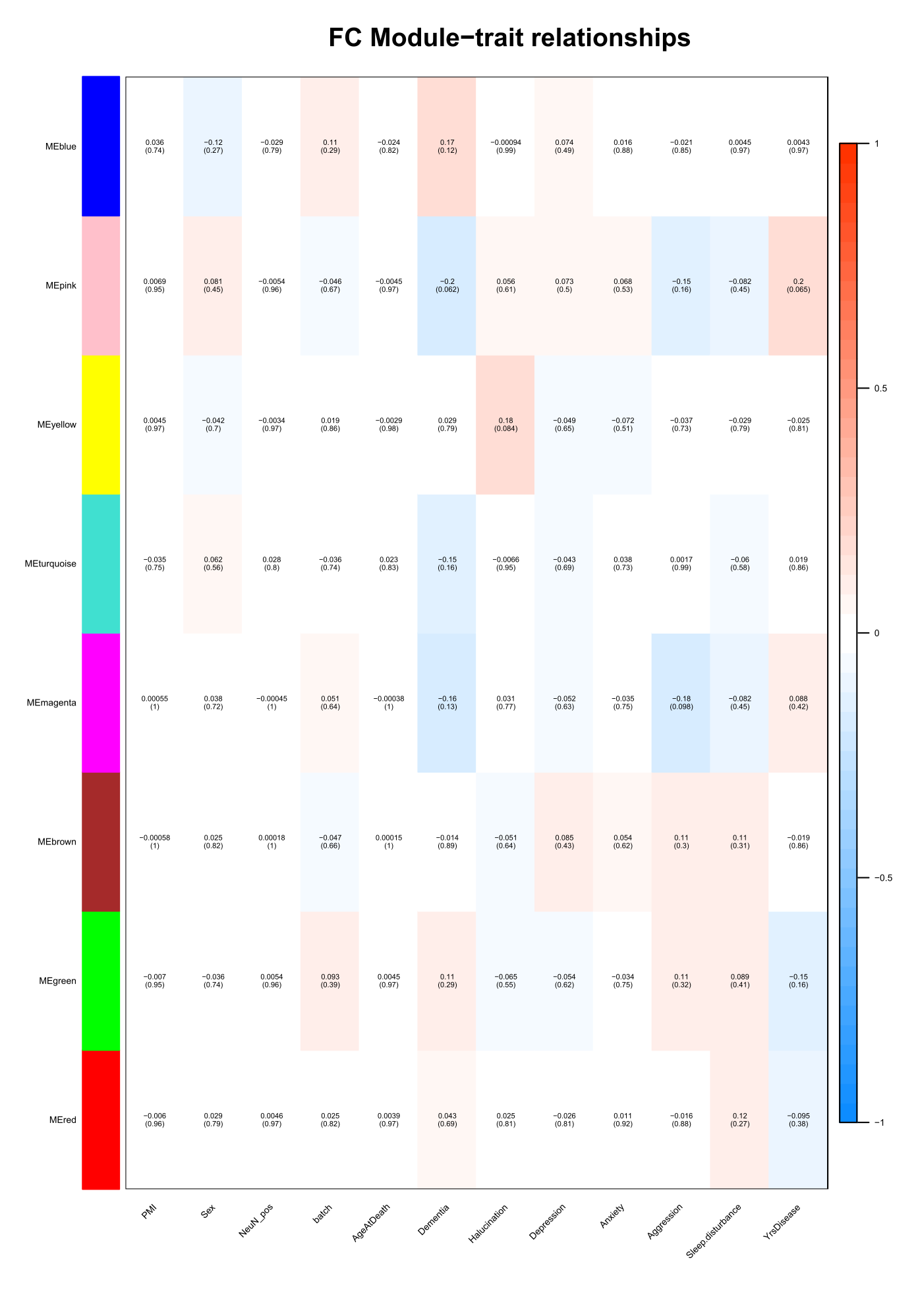
Supplementary Figure 3: Trait-module correlation matrices for the frontal cortex.** Correlations are shown between module eigengenes and traits of interest, with module names (arbitrarily assigned colors) shown along the Y-axis. Correlation estimates are reported, with p-values in parentheses. Grids are colored by correlation estimates. Abbreviations: PMI: Post mortem interval, NeuN_pos: Predicted NeuN+ proportion, batch: Processing batch value, AgeAtDeath: Years of age at death, YrsDisease: Years between diagnosis of PD and death.

**
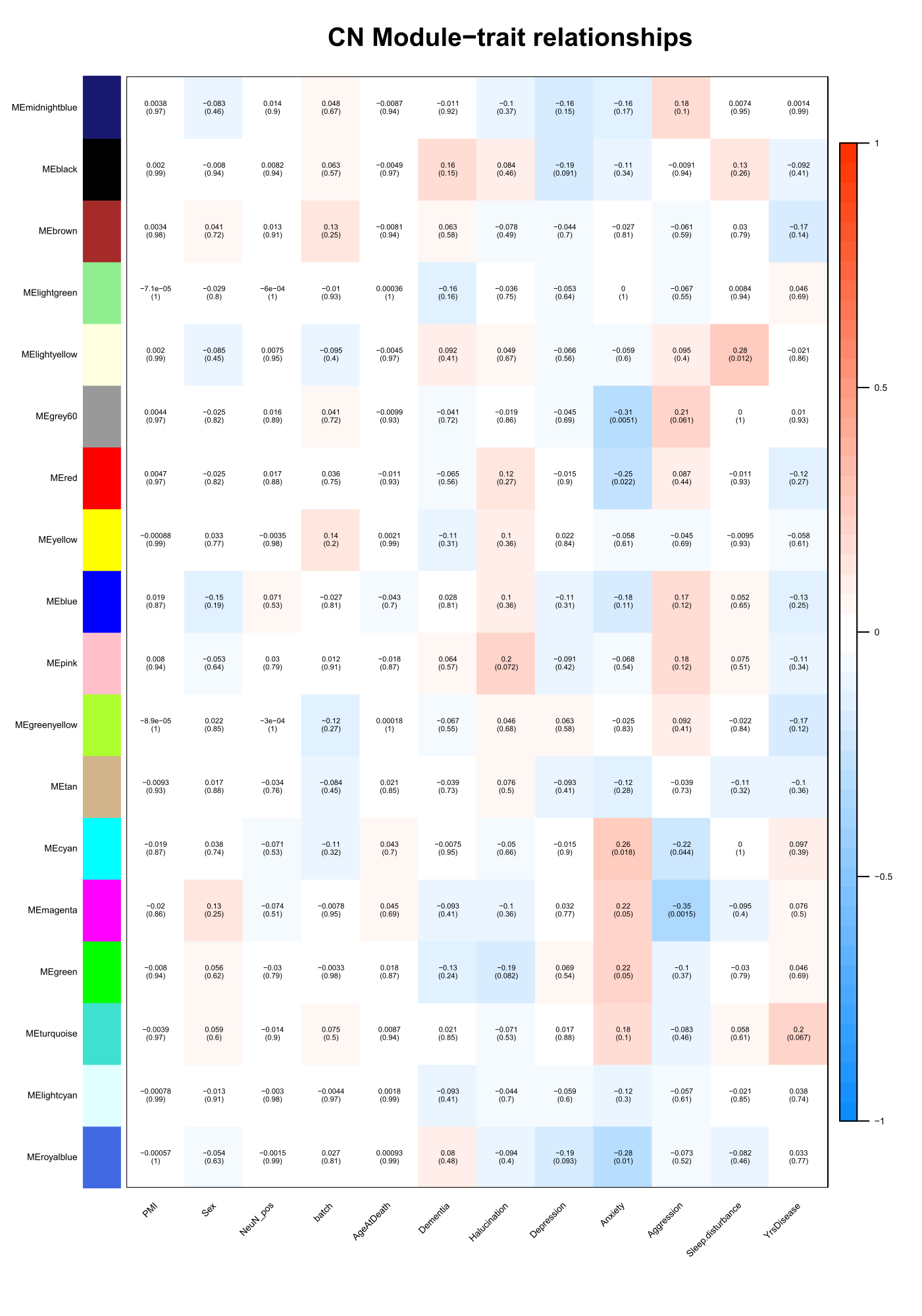
Supplementary Figure 4: Trait-module correlation matrices for the caudate nucleus.** Correlations are shown between module eigengenes and traits of interest, with module names (arbitrarily assigned colors) shown along the y-axis. Correlation estimates are reported, with p-values in parentheses. Grids are colored by correlation estimates. Abbreviations: PMI: Post mortem interval, NeuN_pos: Predicted NeuN+ proportion, batch: Processing batch value, AgeAtDeath: Years of age at death, YrsDisease: Years between diagnosis of PD and death.

**
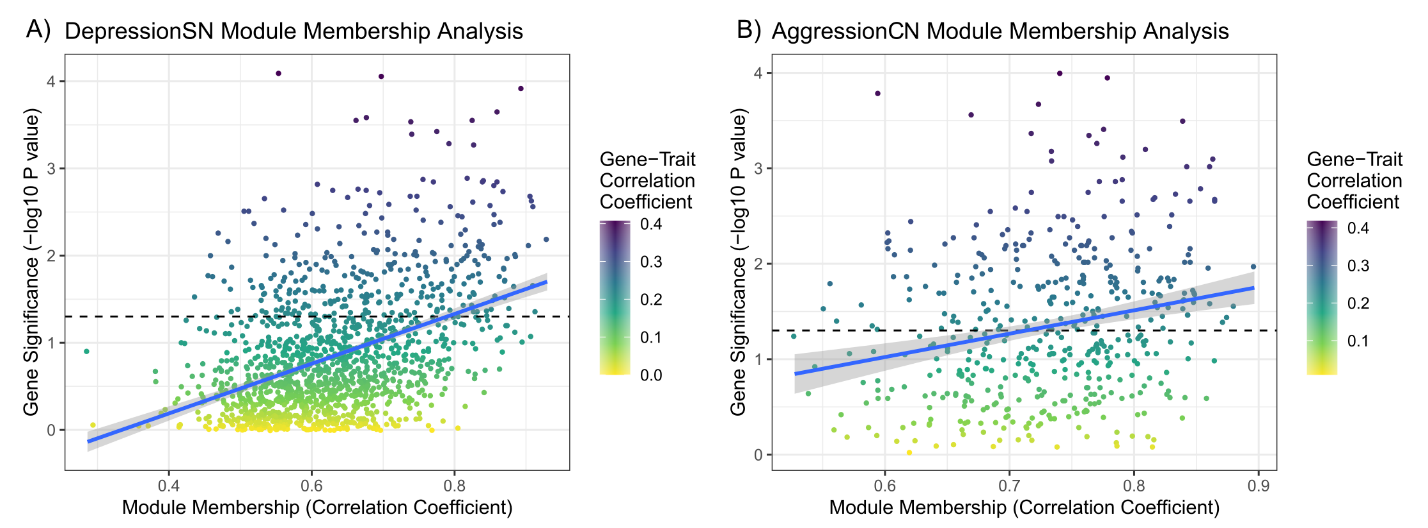
Supplementary Figure 5: Correlation between module membership and gene significance for trait tested.** Module membership (here measured as the correlation coefficient between the individual probes corrected methylation value and the module eigengene), and the gene significance (-log10 transformed p-value) from Spearman’s correlation between each probe and the trait being tested. Measurements are calculated for all probes within the **A)** DepressionSN module (n = 1,375 probes, Pearson’s Correlation Coefficient = 0.12, p-value = 1.24e-05) and **B)** AggressionCN module (n = 475 probes, Pearson’s Correlation Coefficient = 0.07, p-value = 0.13).

**
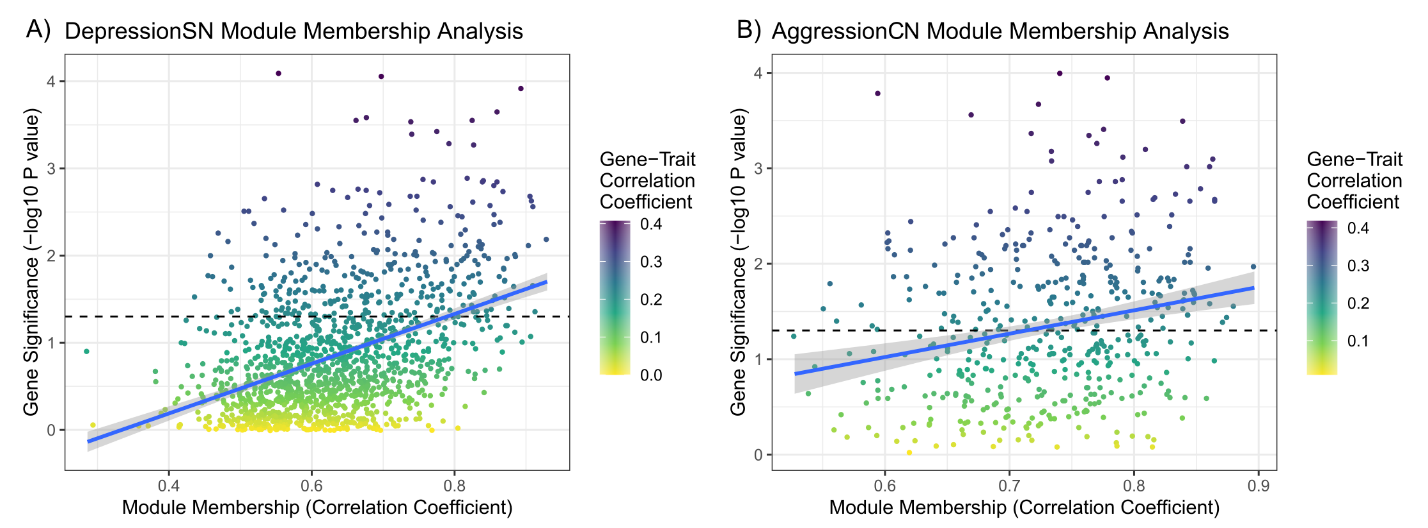
**

**
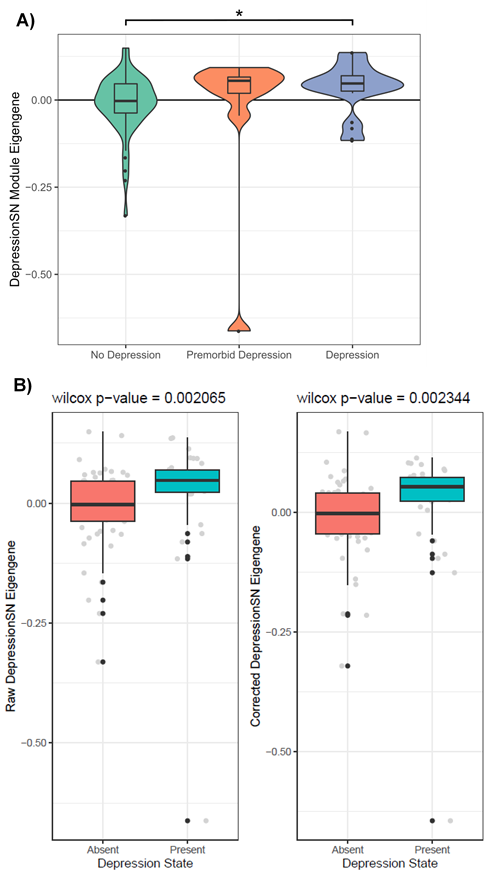
Supplementary Figure 6: A) Violin and boxplots of depressionSN module eigengene compared to depression onset.** Groups are subset by temporal annotation of depression symptoms in the clinical notes. Premorbid depression is a history of depression preceding the primary PD diagnosis. Depression is a group with no annotated history of depression before the primary PD diagnosis. Significance annotated for a pairwise Wilcox rank-sum comparison between each group with BH-correction. * indicates q-value < 0.05 **B) Jitter and boxplots of depressionSN module eigengeneby depression status.** Left hand plot shows raw eigengene values, right hand shows eigengene corrected for Braak Lewy body stage, Braak neurofibrillary tangle stage and years of disease using residuals from a linear model. Wilcox rank-sum test comparisons shown above each plot.

**Supplementary Figure 7: Expression Weighted Cell Type Enrichment Results for all modules detected in the substantia nigra.** Results are displayed as a binary matrix format, with cells coloured based on Benjamini-Hochberg (BH) significant correct p-value for significant enrichment correcting for all 252 separate tests. Modules are shown along the Y-axis and cell types tested for significant enrichment are shown along the X-axis. Highlighted with a black box is the magenta module, corresponding to the DepressionSN module

**
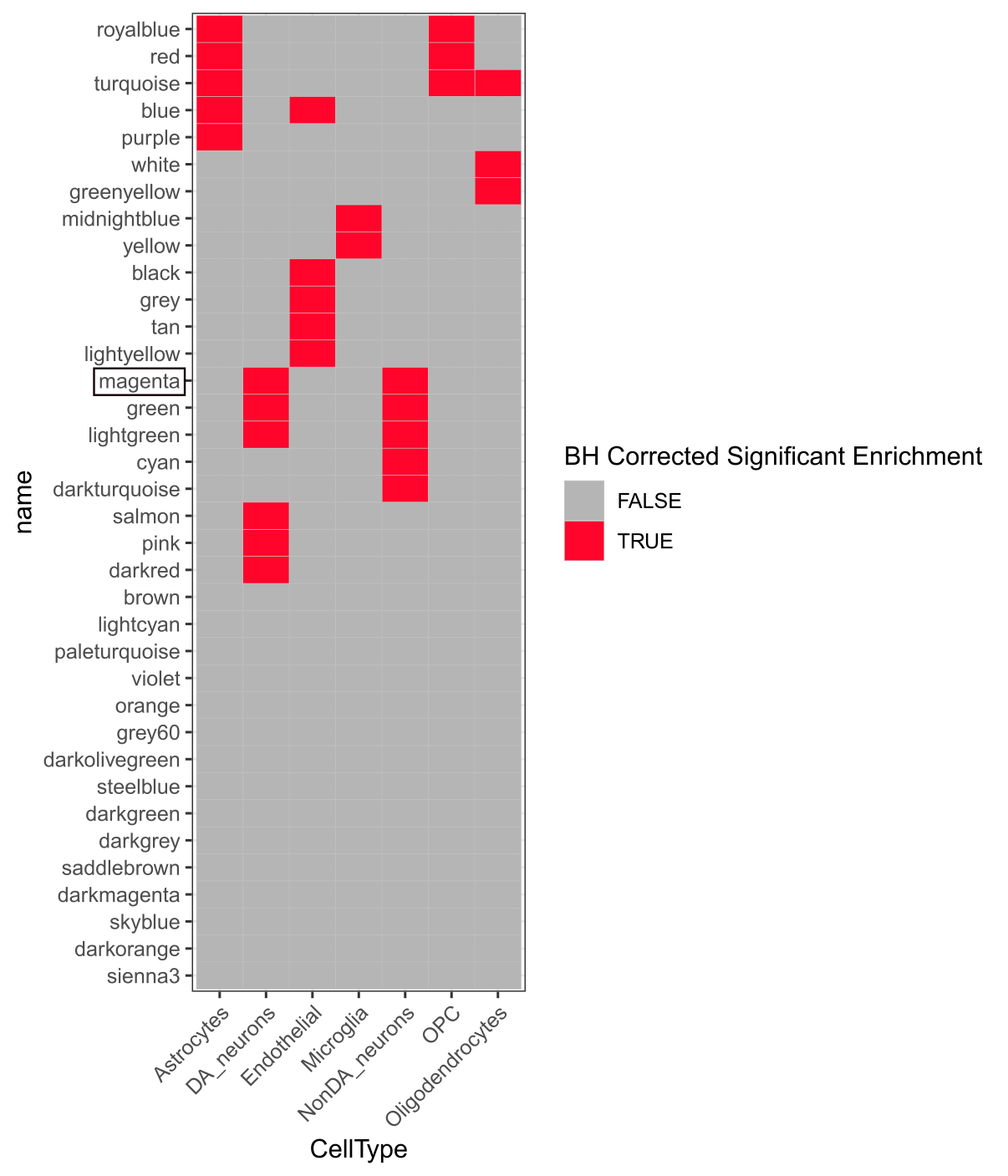
**

**Supplementary Figure 8: Gene Ontology Enrichment plots.** The top 10 most significant terms for gene ontology (GO) enrichment analyses in **A)** the SN Depression associated module and **B)** the CN Aggression associated module. The term titles are displayed along the Y-axes, with -log10(P) for enrichment significance shown along the X-axis. Points are sized by the proportion of the overall ontology gene numbers represented in that specific module

**
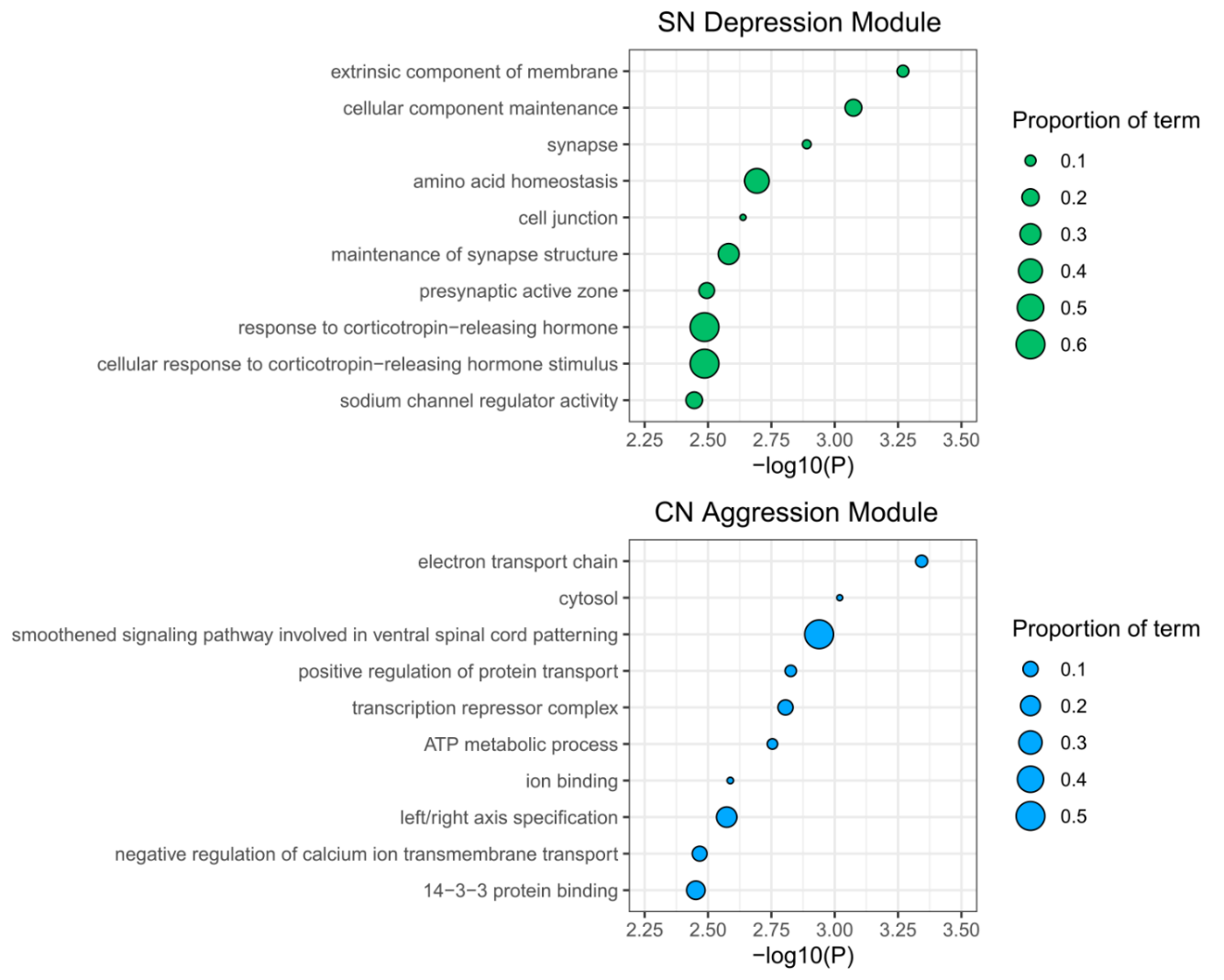
**
